# Fluoroscopic control of a magnetorobotic capsule for precision gastrointestinal sampling and delivery

**DOI:** 10.1016/j.isci.2026.115007

**Published:** 2026-02-11

**Authors:** Sophie Nguyen, Tuan-Anh Le, Melek Naz Guven, Carol Lu, Husnu Halid Alabay, Hakan Ceylan

**Affiliations:** 1 Department of Physiology and Biomedical Engineering, College of Medicine and Science, Mayo Clinic, Scottsdale, AZ 85259, USA

**Keywords:** Drug delivery system, Engineering

## Abstract

Untethered gastrointestinal (GI) devices for targeted diagnostics and interventions are constrained by limited capabilities for precise localization, controlled delivery, and reliable retrieval of liquid or gel-based payloads. Here, we introduce G-Bot, a magnetorobotic capsule that integrates fluoroscopic visibility, dual-regime magnetic actuation, and a low-cost, scalable design. The proposed actuation strategy enables decoupled orientation control and on-demand triggering of payload release or sampling. G-Bot provides sufficient radiographic contrast to support accurate manipulation under standard clinical C-arm. Device performance is evaluated in benchtop GI phantom models using samples spanning a range of viscosities under clinically relevant magnetic conditions, demonstrating image-guided sampling, targeted delivery, and feasibility for longitudinal GI monitoring.

## Highlights

• Fluoroscopy-visible robotic capsule enables precise targeting in the gastrointestinal tract
• Dual magnetic regimes separate orientational control from release and sampling triggers
• On-demand liquid and gel sampling and release are demonstrated without premature leakage
• Low-cost, electronics-free design supports scalable translation and longitudinal studies

## Graphical abstract

**Figure:**
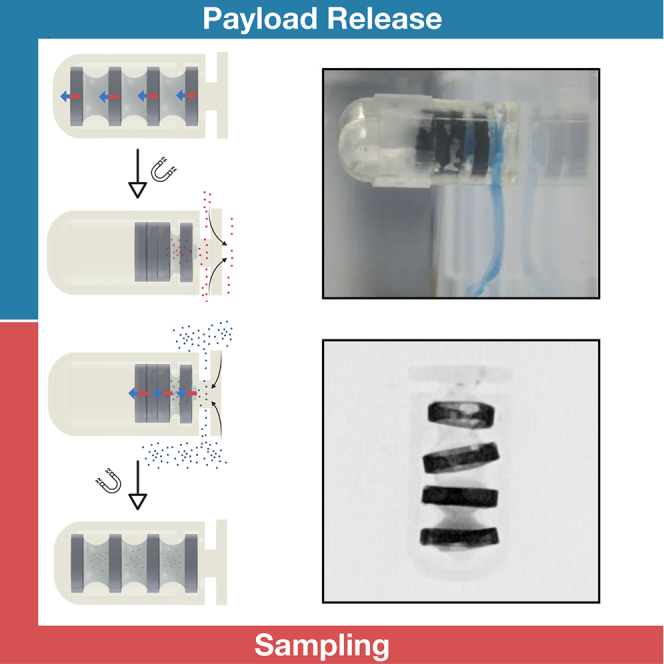

## Introduction

Traditional endoscopies and colonoscopies have been minimally invasive techniques for examining the lower gastrointestinal (GI) tract for decades. Despite their effectiveness, they come with significant drawbacks for the patient, such as discomfort, the need for sedation or anesthesia, and potential risks of complications such as bowel perforation, bleeding, and even death.^1^^,^^2^ Consequently, new approaches are emerging that focus on entirely non-invasive diagnostic applications.

Capsule endoscopy represents one of these innovations, offering an orally ingestible GI imaging system alternative that does not require sedation and can image most of the bottom GI tract.^3^ Companies such as Medtronic and Endiatx have developed capsule endoscopes that are battery-powered and equipped with cameras to transmit real-time images of the GI tract to physicians.^4^ Traditionally, these capsule endoscopes rely on the body’s natural peristaltic movements to travel through the digestive system.

However, Anx Robotica has developed the NaviCam Stomach Capsule System, which can be maneuvered by the practitioner using magnetic control, allowing for more comprehensive imaging of the GI tract.^5^ Swain et al. introduced a neodymium (NdFeB) magnet component to the camera capsule, demonstrating that external magnetic fields can precisely control and localize the capsule within the stomach and esophagus.^6^ NdFeB, when coated, has shown to have an acceptable cytotoxicity range, making it a suitable magnetic material for bioengineering applications.^7^

Building on these advancements, Park et al. applied magnetic actuation for real-time tissue sampling in the GI tract, using magnetic fields to assist with alignment and deployment of a capsule sampling tool. This propulsive technique has been successfully demonstrated for *ex vivo* small intestine and *in vivo* stomach samples, providing an alternative to traditional stool analysis, which is limited to colon samples.^8^^,^^9^

Untethered soft robotics can also be applied to sampling by utilizing magnetic actuation as well.^10^^,^^11^^,^^12^^,^^13^^,^^14^ Dong et al. developed soft capsules with magnetic valves that can be controlled remotely for fluid sampling as needed.^15^ Sun et al.’s MagCaps further illustrate the utilization of magnetically actuated valves for potential drug release.^16^

Capsule platforms can also be used in longitudinal monitoring of chronic illnesses. For example, the Pillcam SB3 system has been used to monitor Crohn’s disease and showcased a strong correlation with ileocolonoscopy scores and was able to identify inflammation that ileocolonoscopy did not detect; moreover, there were no adverse effects from this methodology, establishing capsule endoscopy as a valid and reliable method of evaluating chronic illnesses.^17^ However, many of these current capsule endoscopic systems are still costly, establishing a barrier to diagnostic care.

As designs become more refined and cheaper, endoscopies and colonoscopies can become more accessible, improving patient outcomes and moving the field of medical robotics further. These capsule endoscopy procedures are most likely to be more cost-effective in comparison to traditional endoscopies with increased invasion and need for anesthesia.^18^

These designs could also be adapted for active therapeutic interventions. An algae-based robot, by Li et al., was developed to remove cytokines *in vitro* to treat inflammatory bowel disease models in mice.^19^ This illustrates that the future of robotic capsules is geared toward increasing functionality in both sampling and therapeutic interventions. These capsules could then be applied to active delivery of therapeutics by learning from current sampling mechanisms.

From the current literature, there is still no capsule platform that combines untethered, actively triggered liquid delivery and sampling, magnetic navigation, and direct compatibility with existing fluoroscopy infrastructure. Magnetically steered capsules primarily focus on imaging and localization without on-demand cargo exchange, while recent soft magnetic capsules demonstrate liquid sampling or release but rely on endoscopic visualization or external cameras and do not explicitly decouple steering and actuation thresholds or address clinical X-ray workflows. As a result, there remains a need for a cost-effective, magnetically controllable capsule that can be precisely localized within the lower GI tract, selectively triggered to deliver or retrieve liquid payloads, and readily integrated into current interventional suites.

Here, we introduce G-Bot, a simple and low-cost magnetorobotic capsule endoscope for the lower GI tract that enables local delivery and sampling of liquid cargo. Our specific contributions are: (i) a soft-rigid capsule architecture with a foldable elastic-NdFeB inner pump and 3D-printed outer shell that achieves five-degree-of-freedom magnetic orientation control and on-demand volumetric compression/expansion; (ii) a two-regime magnetic actuation strategy in which low, near-uniform fields provide robust orientation control, while substantially higher field gradients trigger capsule collapse and suction, thereby decoupling steering from release/sampling and minimizing unintended actuation; (iii) quantitative demonstration that the NdFeB-loaded core provides fluoroscopic contrast exceeding clinical 30% iohexol benchmarks, enabling real-time C-arm-guided localization, delivery, and sampling in a phantom under radiation doses below common GI fluoroscopic procedures; and (iv) identification of a materials and geometry combination that offers reliable payload containment without leakage and can be manufactured from low-cost components, supporting repeated deployments in longitudinal studies. This platform may enable targeted sampling, spatiotemporal mapping, and engineering of gut microbiota, as well as localized drug delivery to lesions in inflammatory bowel disease and related disorders.

## Results

### G-bot design and fabrication

**Figure 1:**
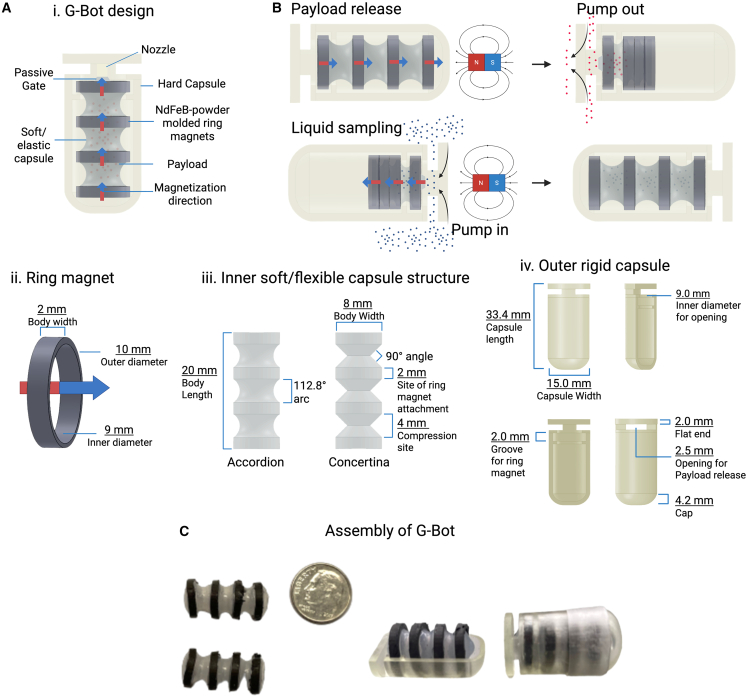
G-Bot design and components (A) (i) Structural schematic of components, (ii) soft inner magnet, (iii) inner flexible capsule structure, and (iv) outer rigid hard capsule dimensions. (B) Responsive liquid cargo delivery and sampling mechanism. (C) Assembly of functional G-Bot.

A G-Bot consists of an inner soft magnetic capsule enclosed within a rigid protective shell made of biocompatible polymer resin (Figure 1A). The soft capsule features four concentrically arranged ring magnets connected via a thin Ecoflex elastomer (Smooth-On). The soft capsule structure was fabricated in two distinct geometries, accordion and concertina, to evaluate folding efficiency and payload release effectiveness (Figure S1). The Accordion design features curved folds, whereas the Concertina design has defined sharp 90° angle folds (Figure 1A). These geometries were chosen to further explore elements of design for optimal payload volume and release.

Each ring magnet consists of NdFeB powder embedded in the Ecoflex mold (1:4 mass ratio) and is magnetized perpendicular to the plane (out-of-plane direction) and arranged in an attractive dipole mode. NdFeB microparticles mixed with Ecoflex formed the magnetic rings. NdFeB was selected for its strong magnetic properties and high coercivity, ensuring stable magnetization under external fields. Higher concentrations of NdFeB were impractical due to increased viscosity and mixing difficulties.

Without an external magnetic field, these magnets alone do not produce sufficient force to induce capsule folding. However, under a strong external magnetic field gradient, the rings collapse inward, creating pressure that pushes the liquid payload through the capsule’s nozzle (Figure 1B). Conversely, when the external field direction is reversed, the repulsion force pushes the ring magnets, creating a suction force into the pump, which drives the sampling of the liquid surrounding.

A passive gate, formed naturally during fabrication by trimming excess polymer, allows payload release only when the inner capsule is actively compressed (Figure S2). Under normal conditions, the gate remains closed because the elastic Ecoflex body applies a restoring force that maintains the nozzle in a sealed configuration. Opening the gate requires the characteristic radial inward collapse of the magnetic rings, a deformation that occurs exclusively under high-gradient magnetic actuation.

The rigid outer capsule further shields the soft core from external mechanical forces and prevents premature deformation or leakage, while also isolating the NdFeB microparticles. Importantly, typical gastrointestinal peristalsis generates predominantly axial and circumferential squeezing rather than radial inward collapse; therefore, physiologic motion is not expected to trigger unintended release. The capsule shell also includes a positioning notch for alignment and a flat end that protects the gate region, and a thin plastic band connects the two-halves for easy assembly (Figure 1C).

In fabricating the soft capsule, two Ecoflex variants were used: Ecoflex 00–10 (Eco10) and Ecoflex 00–50 (Eco50). Eco10 is a more flexible elastomer compared to Eco50; however, Eco50 exhibits greater durability, as evidenced by its higher tensile strength and greater elongation at break.^20^^,^^21^ After observations with the fabrication of each type of Ecoflex, a combination of both was desired. Alone, each was difficult to work with and prone to tears. Thus, the body was fabricated with two layers of Ecoflex and is labeled accordingly. These layered composites were identified by their inner and outer layers: e.g., “Eco50/10” indicates an inner Eco50 layer and an outer Eco10 layer.

The Ecoflex polymer type for each layer had an impact on the thicknesses of those layers. In particular, Eco10/10 and Eco10/50 have the most significant differences in layer thickness between the first coat and second coat of Ecoflex, regardless of the mold geometry used to create the inner soft capsule (Figure S2). However, the other two combinations, Eco50/10 and Eco50/50, were found to have no significant differences in layer thickness for either geometry, showcasing that the adhesion of the silicon polymers to the mold and other cured silicon polymers differs based on silicon type.

To characterize the mechanical behavior of the different G-Bot body constructs under tensile and compressive loading, we performed stretching and compression tests using a CellScale UniVert Universal Testing System (Figure S3). Eco10/50 bot body required less force than the other combinations when stretched at all four extensions: 50%, 100%, 150%, and 200% of the G-Bot body length (Figure S4). This could be used to show increased flexibility and durability for use compared to other combinations. Compression testing was also performed for the four combinations, with the Eco10/10 bot body requiring the least amount of force to compress to 20% of its body length. However, the force required for achieving maximal compression (around 80%) was comparable for all four G-Bot body variations, ranging from 0.54 N to 0.70 N (Figure S5). These forces are compatible with our exact magnetic actuation capacity needed to trigger compression or expansion of the ring magnets.

### Actuation control

Magnetic actuation of the G-Bot operates through two decoupled regimes.

1. low-field orientation control and
2. high-gradient compression for payload release or sampling.

**Figure 2:**
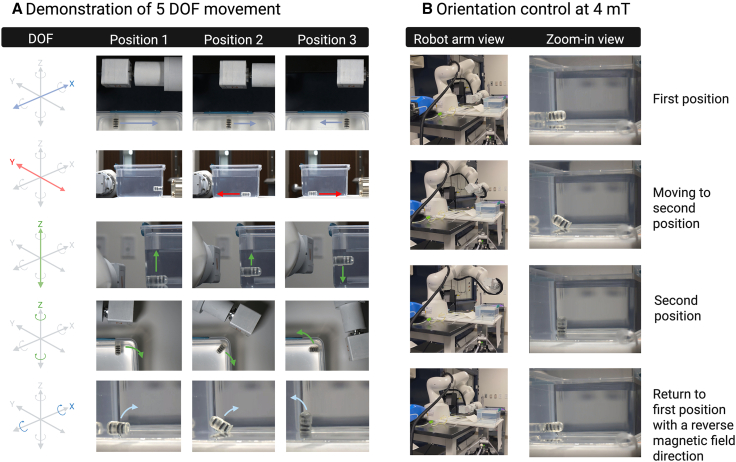
G-Bot orientation control using external magnetic fields (A) 5 DOF control and (B) a prescribed path for orientation control under 4 mT.

Orientation is achieved by aligning the magnetic moment of the capsule with the external field using magnetic torque, defined by the equation:

T=m×B

where *T* is the magnetic torque, *m* represents the total magnetic moment vector of the inner capsule, created by the ring magnets, and *B* is the external magnetic field vector applied to the G-Bot. To experimentally demonstrate orientation control, a cylindrical permanent magnet (76 mm × 76 mm × 38 mm, N52 NdFeB) was mounted onto a robot arm (LBR Med 7 R800, Kuka).^22^ This magnet was positioned at varying distances from a water-filled container housing the G-Bot with an accordion-shaped inner capsule. The capability for orientation control with 5° of freedom was demonstrated (Figures 2A and S6, and Video S1).

A near-uniform field of 4 mT (gradient 0.1 T/m) produced sufficient torque for reliable alignment without inducing collapse of the soft magnetic rings (Figures 2B and S7). At larger magnet distances, torque dominated over translational force, enabling orientation without net motion toward the magnet. This establishes a clinically relevant steering regime, as 4–10 mT fields can be generated at 10–30 cm, within typical abdominal thickness.

Video S1. Demonstration of five-degrees-of-freedom orientation control, related to Figure 2

Following alignment, payload exchange requires radial inward collapse of the magnetic rings, driven by field gradients according to

F=(m·∇)B

where F is the magnetic force acting on the ring magnets.

The magnetic moment of each ring magnet was calculated from the volume of NdFeB in one ring (approximately 1.08× 10^−8^ m^3^) and magnetic flux density (0.47 T), resulting in a magnetic moment of 4.04× 10^−4^ A·m^2^ (Supplementary Text 1).^23^ Simulation in COMSOL Multiphysics showed that at 10 mm distance from the external magnet, the G-Bot experienced magnetic fields ranging from 131 to 192 mT across the rings, corresponding to gradients of 7.3–10.7 T/m and generating a maximum magnetic force of approximately 112 mN (Figures S7 and S8). Reducing the distance to 5 mm increased the external magnetic field strength to 260 mT with gradients reaching ∼13.2 T/m and raised the maximum force to 152 mN (Figure S7). These forces illustrate the impact of an external magnetic gradient in triggering the compression of the G-Bot.

These simulation results were further verified via magnetic force measurements with an experimental setup (Figure S9). Using the robot arm external magnet, the G-Bot’s magnetic forces were measured for a vertical and lateral configuration across 5 mT–130 mT (corresponding to magnetic field gradients of 0.1 T/m to 7.3 T/m). In the vertical configuration, the maximum force was 127.3 mN at 130 mT, with a pressure calculated to be 720 Pa for the contact surface area (1.77 cm^2^). In the lateral configuration, the maximum force was 151.3 mN at 130 mT, with the pressure calculated to be 192 Pa for the lateral contact surface area (7.87 cm^2^). These pressures are significantly below the reported small intestine burst pressure (0.81 MPa), confirming that the magnetic actuation forces are safe and adequate for payload delivery without causing damage.^24^^,^^25^

### Payload release and leak test

**Figure 3:**
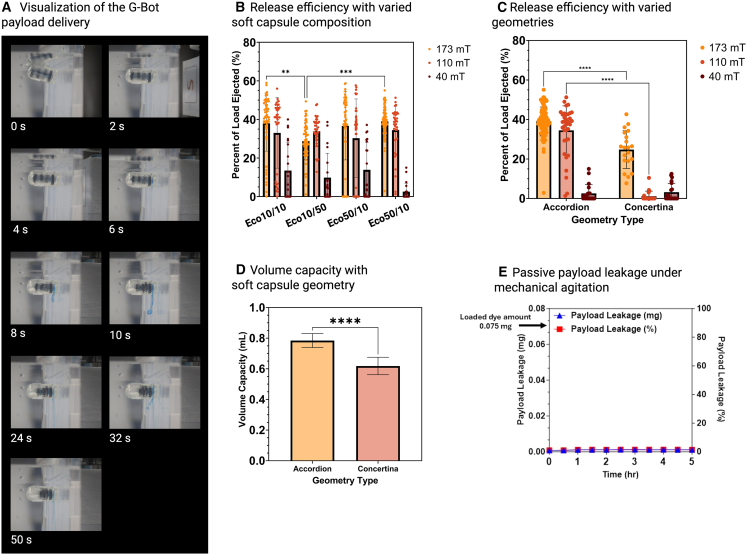
Controlled payload release with magnetic trigger (A) Visualization under an external 108 mT magnetic field triggering the payload release. (B) Release efficiency refinement using various elastomer compositions for the inner soft capsule. (*n* = 20 for each group; one-way ANOVA) (C) efficiency for accordion and concertina models at 173 mT (*n* = 20 for each group; one-way ANOVA), (D) volume capacity for the accordion and concertina models (*n* = 60 for each group, two-tailed Student’s *t* test), and (E) Dynamic leakage test of the G-Bot under continuous agitation. (For each variation, ∗∗∗∗ denotes a *p* value <0.0001, ∗∗∗ denotes a *p* value between 0.001 and 0.001, ∗∗ denotes a *p* value 0.001–0.01, and ∗ denotes a *p* value 0.01 to 0.05.) All data are represented as mean ± standard deviation.

A minimum of 108 mT with a magnetic gradient of 5.4 T/m (as measured on a Gaussmeter) is needed from the permanent magnet to trigger the release of the payload, driven by the compression and the push-out of the payload from the robot nozzle (Figure 3A and Video S2). The efficiency of payload release was tested with various inner capsule designs and at three different magnetic field strengths: 173 mT (magnetic field gradient of 8.7 T/m, 108 mT (magnetic field gradient of 5.4 T/m), and 40 mT (magnetic field gradient of 1.5 T/m) (Figure S7). These magnetic fields were generated by placing the robot arm magnet at various distances from the G-Bot and observing the amount of payload released immediately after compression for 30 min.

Video S2. Demonstration of controlled release with magnetic actuation, related to Figure 3

Beyond 108 mT, no significant difference was found between the release efficiencies of the different G-Bot based on their polymer configuration (Figure 3B). While there were significant differences in some release efficiencies at 173 mT, there was no singular model that had a significantly higher release efficiency than the other configurations at all three magnetic field strengths. Moreover, with payload delivery, the G-Bot compresses to 66% of its body length (34% compression). From Figure S5, approximated at below 0.1 N, is necessary to achieve the 34% compression seen in payload delivery for all G-Bot configurations. This verifies the use of the above external magnetic field strengths in triggering payload delivery.

Next, two primary G-Bot inner soft capsule geometries, the Concertina model and the Accordion model, were compared to optimize release. It was found that the Accordion model had a significantly higher release efficiency at 173 mT and 110 mT (Figure 3C). There were also no significant differences between the release efficiency for both geometries at 40 mT. Additionally, the Accordion model also had a greater volume capacity than the Concertina model (Figure 3D). These findings demonstrate how the Accordion design’s curved folds enabled greater internal compression and payload capacity, compared to the Concertina design’s defined, sharp 90° angle folds (Figure 3).

Moving forward, the G-Bot accordion model Eco50/50 was chosen to be the lead design due to its most reliable release efficiencies at 40 mT (Figure 3B). With a low G-Bot responsiveness at 40 mT, the magnetic strength difference needed for orientation control and delivery increased, allowing for an increased buffer range before triggering delivery in case of accidentally jostling the G-Bot. The significant magnetic strength difference needed between orientation control and delivery allows for successful decoupling of the functions.

With this design, passive release was also tested through a leak test. G-Bot with 0.0075 mg methylene blue dye payload (0.01 mg/mL) in 50 mL water was placed on an orbital shaker at 50 rpm in the absence of a magnetic field. The resulting change in the absorbance samples from the surrounding environment over 5 h was used to determine the amount of payload passively released from the gate (Figure 3E). From Figure 3E, it can be seen that less than 1% of the payload (within the margin of sensitivity) was passively released over the 5-h period, indicating that uncontrolled leakage is minimal and that the passive gate remains effectively sealed without magnetic actuation. These findings confirm that the G-Bot enables highly controlled, targeted payload release only upon the application of an external magnetic field.

### Fluoroscopic visibility of G-bot and sampling

**Figure 4:**
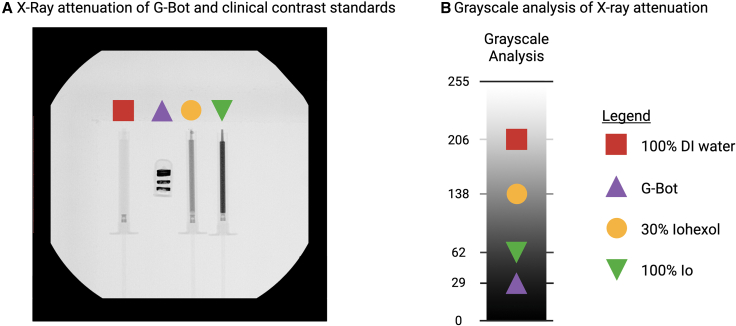
Clinical fluoroscopic visibility of G-Bot (A) The X-ray attenuation of a G-Bot compared to 1-mL filled syringes of varying iohexol concentrations, clinical standard of Omnipaque 350. (B) Grayscale analysis of the fluoroscopic image shows relative contrast. 30% iohexol sets the lower boundary of iodine concentration that offers visibility within the human body.

Achieving sufficient contrast is critical for detecting the robot while managing patient radiation exposure. To ensure the compatibility of G-Bot with clinical C-arm systems, its radiographic contrast was quantified relative to 1-mL standard syringes of varying concentrations of an iodine-based medical contrast agent, iohexol, specifically its endovascular formulation, Omnipaque 350 (GE Healthcare), as the clinical benchmark. For fluoroscopy-guided interventions, a medical device should exhibit contrast levels at least equivalent to 30% iohexol to ensure adequate visibility. Grayscale analysis of the samples revealed that G-Bot exceeded both 30% and 100% iohexol thresholds, indicating that it would be readily visible in a clinical setting (Figure 4). This superior radiographic visibility results from the higher atomic number of _60_Nd within NdFeB magnetic powder compared to _53_I, which increases X-ray attenuation and thus enhances detectability under a C-arm.

**Figure 5:**
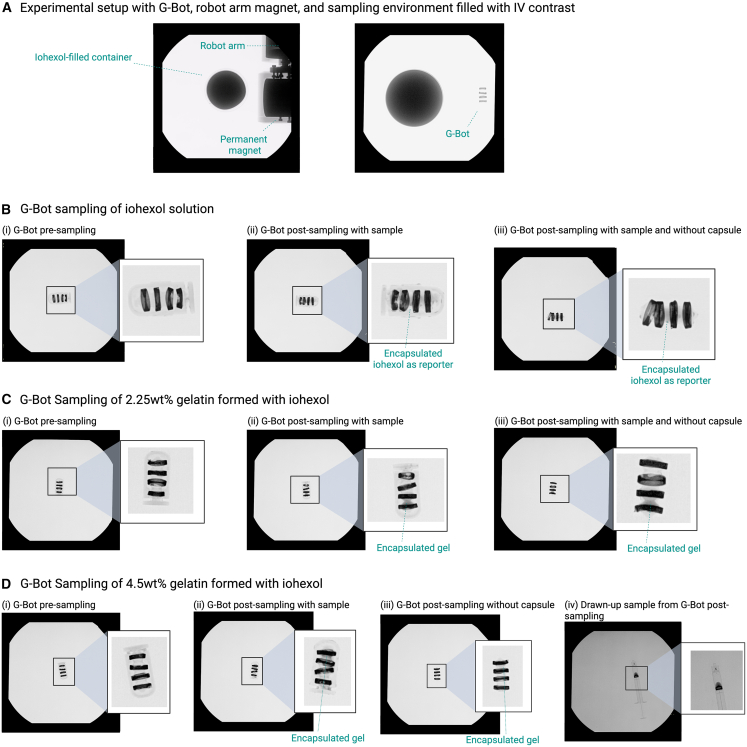
Sampling demonstrations under fluoroscopic imaging, where uptake of radiopaque material served as a sampling reporter (A) Experimental field of view shows the G-Bot submerged in 100% iohexol (clinical gold-standard contrast agent, Omnipaque 350 used without dilution) in the presence of a permanent magnet and robot arm. Despite the highly radiopaque environment, the G-Bot remains clearly visible. (B) Sampling of 100% iohexol: (i) pre-sampling radiopaque inner soft core, (ii) post-sampling with contrast visible within the soft core, and (iii) post-sampling after the removal of the outer shell showing retained contrast. (C) Sampling of viscous gelatin solution (2.25 wt %) in 50% iohexol/50% water: (i) pre-sampling radiopaque inner core, (ii) post-sampling contrast visible within the inner core, and (iii) post-sampling after outer-shell removal confirming contrast uptake. (D) Sampling of highly viscous gelatin (4.5 wt %) in 50% iohexol/50% water: (i) pre-sampling radiopaque inner core, (ii) post-sampling with contrast visible within the soft core and along the inner capsule wall, and (iii) post-sampling after outer-shell removal shows contrast localized between the radiolucent rings, (iv) syringe extraction confirming consistent 0.2–0.3 mL of sampled viscous material (*n* = 3 for each group).

The G-Bot was then tested for its sampling capabilities. It was first evaluated in a homogeneous liquid environment containing 100% iohexol (Omnipaque 350), the clinical gold-standard contrast agent, used here without dilution. Iohexol served as a radiopaque reporter to visualize fluid uptake during sampling. Despite the highly radiodense surroundings, the G-Bot remained clearly visible under fluoroscopy due to the radiopaque soft core and radiolucent ring magnets (Figure 5A). A switching external magnetic field was subsequently applied to compress the soft inner core and then draw in surrounding fluid. Pre-sampling imaging showed a uniformly radiopaque inner core with radiolucent rings (Figure 5B i). Following actuation, distinct regions of radiolucency appeared within the inner core and between the rings (Figure 5B ii–iii), indicating successful uptake of the contrast medium. These results demonstrate that the G-Bot can reliably sample its liquid environment using alternating magnetic field polarities.

Sampling performance was then evaluated in more challenging viscous environments using 2.3 wt % and 4.5 wt % gelatin mixtures prepared in a 50% iohexol and 50% deionized water solution. The 2.3 wt % gelatin mixture represents a moderately viscous fluid, while the 4.5 wt % formulation produces a highly viscous, gel-like medium that more closely approximates the physical behavior of mucoadhesive or gel-based therapeutics. In both cases, the radiopaque iohexol served as a reporter for visualizing fluid uptake within the G-Bot.

As in the liquid testing, the G-Bot was introduced into each gelatin environment and actuated using a switching external magnetic field to compress the soft inner core and subsequently draw in the surrounding material (Figures 5C and 5D). For the 2.3 wt % gelatin solution, fluoroscopy revealed a clear increase in radiolucency within the inner core relative to the pre-sampling state, both with the capsule intact and after removal of the outer shell (Figure 5C). Syringe extraction across three independent trials confirmed a repeatable sampled volume of 0.2–0.3 mL.

For the highly viscous 4.5 wt % gelatin, fluoroscopy suggested successful uptake within the soft core, though portions of the sample were obscured by the extremely radiolucent ring magnets (Figure 5D ii-iii). To resolve this, the G-Bot was imaged using 3D C-arm fluoroscopy. Reconstruction using 3D Slicer and Fusion360 revealed approximately 0.3 mL of material within the inner core, which was consistent with syringe extraction measurements (Figure 5D iv).

Across both viscous and highly viscous conditions, the G-Bot demonstrated a repeatable sampling capacity of 0.2–0.3 mL, confirming its ability to acquire material even in gel-like media with substantially increased mechanical resistance.

### Application demonstration in a phantom system

**Figure 6:**
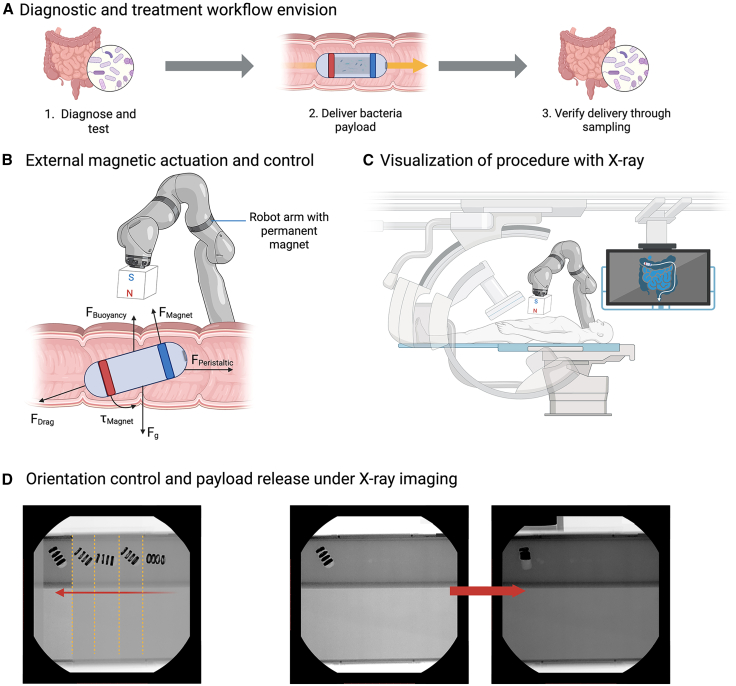
Clinical vision of the applications of soft robotics in the GI tract^26^ (A) After diagnosing the bacterial imbalance, the G-Bot would be administered to deliver the needed bacteria. Then, sampling would be done to verify the delivery efficacy. (B) A robot arm would be used to externally control the G-Bot within the GI tract as it faces various forces, such as the GI tract’s natural peristaltic motion. (C) During the procedure, the G-Bot would be visualized using a C-arm X-ray machine to ensure the precise location of the bacterial payload. (D) Orientation control and payload release of G-Bot under imaging guidance.

The overall clinical vision of G-Bot was summarized in Figures 6A–6C. As a prerequisite for utilizing the G-Bot, the patient’s bacterial deficiencies must first be diagnosed. After, the soft G-Bot will be administered to deliver the bacteria payload while being controlled and operated using an external magnetic field. Fluoroscopic guidance will be used to provide real-time spatial feedback of the G-Bot within the GI tract. After the procedure and appropriate growth time for the bacteria, further testing and sampling will be necessary to verify the effectiveness of the delivery.

In accordance with the clinical vision, a test was conducted to combine both functionalities to showcase the G-Bot’s capability of navigating an environment similar to the GI tract and delivering to the correct location with fluoroscopic guidance. A silicon tube was coated and placed in an opaque 6 wt % gelatin environment to imitate the viscosity of intestinal chyme. The G-Bot was navigated to the target location along the tube via an external magnetic field. When a stronger magnetic field was introduced, the G-Bot delivered its payload.

Figure 6D demonstrates the movement of the G-Bot throughout a phantom colon model to a specific location for payload delivery, visualized via X-ray fluoroscopy. Throughout the procedure, a total dosage of 0.802 Gy·cm^2^ of radiation was exposed to manipulate the G-Bot into position and induce payload release. In comparison to typical diagnostic X-rays, this dosage is less than the radiation exposure dosages used for kidney and urinary bladder AP images.^27^

From the successful demonstration of this project, further testing can be done to optimize and analyze the applicability of the soft robot design in gastrointestinal conditions.

## Discussion

The primary objective of the G-Bot design was to create an ultra-cost-effective capsule capable of robust, on-demand liquid release and sampling in both low-viscosity and gel-like environments. The demonstrations presented here establish a foundational proof-of-concept and suggest that multiple capsules could be deployed sequentially for longitudinal microbiome mapping, enabling localized interventions and continuous monitoring of gut microbial dynamics.

This work identifies a combination of commercially available materials and optimized soft-rigid geometries that reliably contain liquid payloads while supporting controlled magnetic actuation. We demonstrate a two-stage magnetic actuation regime in which low, nearly uniform fields (∼4 mT; 0.1 T/m gradient) achieve stable orientation control, whereas fields approximately twenty times stronger are required to actuate the soft pump mechanism. This clear separation between orientation and release thresholds prevents unintended actuation and enhances reliability. Furthermore, G-Bot integrates seamlessly with standard X-ray fluoroscopy, operating at radiation levels well within routine clinical imaging ranges, enabling precise real-time localization without specialized sensing hardware.^27^

The ability to deliver payloads with spatial precision has important implications for microbial dysbiosis-related disorders, including inflammatory bowel disease, which affects approximately 2.39 million Americans and lacks targeted microbiota-directed therapies.^28^^,^^29^^,^^30^^,^^31^^,^^32^^,^^33^ Given the spatial heterogeneity of gut microbial communities, precise delivery is essential to avoid off-target bacterial growth, a known risk in interventions such as fecal transplantation.^34^^,^^35^^,^^36^^,^^37^^,^^38^ Normal transit times, 3.3 to 7 h, in the small intestine, and 15.9 to 28.9 h, in the colon, make targeted interventions feasible with proper localization control.^39^^,^^40^

Moreover, from the basis of liquid sampling, the G-Bot could be further improved to achieve comprehensive sampling of the microbiome, allowing easier access to the small bowel and niche sections of the GI tract. This unique localized sampling provides a more realistic sampling of intestinal mucosa, which is not completely captured with current fecal sampling.^41^ In contrast, recent studies have demonstrated that fecal sampling is not representative of intestinal mucosal-associated microbiota and has its own variation between multiple stool samples.^42^^,^^43^ The G-Bot could instead provide that intestinal-specific microbiota sample to improve understanding of certain disease processes, especially as the microbiome makeup varies spatially throughout the entire gastrointestinal tract.

Stool sampling, while non-invasive and widely used, primarily reflects luminal contents and does not reliably capture the mucosal-associated microbiota, which is more closely linked to inflammatory and barrier-related diseases. Because microbial communities vary markedly across intestinal regions, stool provides no anatomical resolution and often shows high variability between bowel movements. A localized sampling strategy such as G-Bot, therefore, offers complementary diagnostic value by enabling site-specific and mucosa-relevant microbiota profiling, which stool-based methods cannot achieve.

A distinguishing feature of G-Bot is its minimalist architecture. Unlike capsule endoscopes that integrate cameras, batteries, communication modules, and power systems, G-Bot operates entirely without electronics. All functionality arises from embedded NdFeB microparticles and the soft mechanical structure, resulting in a device that is inexpensive, scalable, and simple to fabricate. This low-cost nature supports repeated multiple deployments within the same patient for longitudinal monitoring or therapy.

Several next-step developments are required for translation toward preclinical testing. The current prototype (31.2 mm × 15.0 mm) exceeds FDA-approved maximum dimensions for swallowable capsules, making miniaturization a key next step.^44^ While the current prototype delivers and samples a fixed volume defined by the soft-body geometry, the current design may limit its delivery efficiency with payload residue within the chamber or limitations in the expulsion force provided by compression. Thus, future iterations will incorporate graded magnetic actuation and potentially multi-chamber architectures to enable finer dose modulation and controlled partial-volume sampling or delivery.

The accordion/concertina architecture was specifically introduced to increase chamber collapse and reduce dead volume during magnetic compression. However, we still observe residual payload entrapment, which we attribute to a combination of (i) incomplete local collapse of the compliant chamber due to the magnetic ring hindrance, (ii) capillary/meniscus pinning within folds and corners, and (iii) fluid-wall adhesion that limits full evacuation. Thus, while the foldable structure improves repeatability and is promising relative to non-folding designs, complete evacuation remains constrained by fluid-structure interactions rather than chamber compactness alone.

Additionally, long-term durability, biocompatibility, and mechanical integrity under physiological conditions must be evaluated. Although Ecoflex and NdFeB composites show favorable cytocompatibility, the 3D-printed Clear V4 shell is not certified for ingestible medical devices.^45^^,^^46^ Future *in vivo* versions will utilize medically certified polymers such as polycarbonate or polypropylene and undergo full ISO-10993 cytotoxicity, irritation, and extractables/leachables testing to ensure safety.

X-ray fluoroscopy was selected owing to the strong radiopacity of NdFeB, even in the presence of intraluminal gas, where ultrasound imaging is limited. The radiation exposure used here (0.802 Gy·cm^2^) is far below levels reported for fluoroscopy-guided GI procedures such as ERCP (8–333 Gy·cm^2^).^47^ In clinical use, dose-minimizing strategies (pulsed mode, collimation, intermittent imaging, last-image hold) would further reduce exposure. Parallel work will explore magnetic localization and hybrid tracking methods that require only occasional fluoroscopic confirmation.

Additional refinement will be achieved through the re-optimization of the external magnetic actuation as capsule dimensions decrease, as miniaturization directly alters magnetic coupling and available force-torque margins.^44^^,^^48^^,^^49^^,^^50^ Studies in anatomically realistic GI phantoms, incorporating mucosal folds, tissue contact, the absence of buoyancy, and dynamic peristaltic loading, will be essential for evaluating the reliability of navigation and orientation within the intestinal folds. These environments more accurately capture frictional loading and geometric confinement in the digestive tract and represent a critical next step toward validating G-Bot’s performance *in vivo*.

To conclude, G-Bot represents a clinically inspired, low-cost, electronics-free magnetorobotic capsule capable of fluoroscopy-guided liquid delivery and sampling. Its decoupled actuation strategy, strong radiographic visibility, safe magnetic pressure range, and scalable manufacturing provide a promising foundation for precision microbiota sampling, targeted therapeutic delivery, and longitudinal monitoring in gastrointestinal disease. Continued work in miniaturization, biocompatibility, advanced phantoms, and *in vivo* validation will position G-Bot as a transformative technology in soft robotic interventions for precision gastroenterology.

### Limitations of the study

This study has several limitations that should be acknowledged. First, G-Bot validation was conducted in controlled experimental settings that, while appropriate for establishing feasibility and performance, do not fully capture the biological, mechanical, and physiological complexity of *in vivo* environments, including dynamic tissue motion, heterogeneous material properties, and long-term biological responses. Second, the present design and control strategies were optimized for specific dimensional and geometric constraints, and performance across broader anatomical variability and pathological conditions remains to be evaluated. Third, this work focuses on short-term operation and does not address long-term biocompatibility, material durability, or chronic tissue interactions, which will be critical for translational and clinical applications. Finally, although the current fabrication approach enables rapid prototyping and design flexibility, additional efforts will be required to address scalable manufacturing, sterilization compatibility, and regulatory considerations necessary for clinical integration.

## Resource availability

### Lead contact

Requests for further information and resources should be directed to and will be fulfilled by the lead contact, Hakan Ceylan (ceylan.hakan@mayo.edu).

### Materials availability

G-Bot design files are available from the lead contact upon request.

### Data and code availability

• All data reported in this article will be shared by the lead contact upon request.
• This article does not report original code.
• Any additional information required to reanalyze the data reported in this article is available from the lead contact upon request.

## Acknowledgments

H.C. acknowledges financial support for this work from The Opatrny Family Foundation.

## Author contributions

Conceptualization, H.C.; methodology, H.C. and S.N.; investigation, S.N., T.A.L., M.N.G., C.L., and H.H.A.; writing – original draft, S.N. and H.C.; writing – review and editing, H.C., S.N., T.A.L., M.N.G., and C.L.; funding acquisition, H.C.; resources, H.C.; supervision, H.C.

## Declaration of interests

Authors declare no competing interests.

## Declaration of generative AI and AI-assisted technologies in the writing process

During the preparation of this work, the authors used OpenAI ChatGPT 5.1 in order to improve language clarity and organization. After using this tool or service, the author(s) reviewed and edited the content as needed and take full responsibility for the content of the publication.

## STAR★Methods

### Key resources table

**Table:** 

REAGENT or RESOURCE	SOURCE	IDENTIFIER
**Chemicals, peptides, and recombinant proteins**		

Ecoflex 00-10	Smooth-On, Inc.	N/A
Ecoflex 00-50	Smooth-On, Inc.	N/A
Clear Resin v4	Formlabs	Cat# rs-f2-gpcl-04
NdFeB magnetic powder	Magnequench	Cat# MQP-15-7-20065-070
Omnipaque 350	GE Healthcare	N/A

**Software and algorithms**		

LBR Med 7 R800	KUKA Global Robotics	N/A
Medixant RadiAnt DICOM Viewer (version 2023.1, 64-bit)	RadiAnt	N/A
SolidWorks	GoEngineer	RRID: SCR_024908
COMSOL Multiphysics 6.1	COMSOL Inc.	RRID:SCR_014767
BioRender Content	BioRender	RRID: SCR_018361

### Method details

#### Fabrication of the magnetic soft core

A 3D model of the dip mold was created via SolidWorks and printed using a FormLabs 3B+ model 3D printer and Clear V4 photopolymer resin. After the 3D model was printed, it was washed with isopropyl to remove any scaffolding and excess resin. Then, the 3D model was heated under a UV lamp at 60°C for 1.5 hours or until the mold was completely dried. The molds and models were then coated in Ease Release 200 to ensure easy release from the Ecoflex series (Smooth-On, Inc) silicon elastomers in the next step. To fabricate the soft body, equal mass weights of Ecoflex Part A and Ecoflex Part B were combined. Ecoflex 00-10 Parts A and B or Ecoflex 00-50 Parts A and B were used depending on the desired soft body formulation. The solution was then degassed in a vacuum chamber for 10 minutes to prevent any air bubbles when coating the molds. Then, the 3D-printed models were slowly and carefully dipped and screwed into the mixture until the desired areas were fully covered in Ecoflex. The model was then allowed to cure for 4 hours at room temperature. The process was repeated to create a double-coated Ecoflex soft body. The Ecoflex polymer was then removed from the mold using tweezers. The process is visualized in Figure S1.

A similar process was used to fabricate the soft ring magnets. After mixing Ecoflex 00-10 Part A and Ecoflex 00-10 Part B, NdFeB particles (Magnequench) were also added to the mixture at four times the weight of the combined Ecoflex solution so that NdFeB to Ecoflex had a 4 to 1 mass ratio. After degassing the solution, the mixture was then carefully poured over the molds. After curing for 4 hours, the molds were placed into a Yoke magnet with a strength of 1.0 T for 30 seconds to actuate the material. The rings were then removed from the mold. The Yoke magnet design can be found in Figure S1C.

The rings were carefully attached to the Ecoflex soft body with Ecoflex 00-10 as “glue” between the parts with the rings arranged so there was a uniform magnetic direction between the magnetic rings. After curing, the G-Bot was then placed inside a 3D-printed capsule to be tested.

#### Mechanical characterization of the magnetic soft core

Ecoflex 00-50 (Eco50) and Ecoflex 00-10 (Eco10) were used in different combinations to create the double-layered Ecoflex soft body. The Ecoflex layer thickness was measured in between coats using a caliper to understand thickness variations. The Ecoflex combinations tested were: 2 layers of Eco10, first layer of Eco10 with a second layer of Eco50, 2 layers of Eco50, and first layer of Eco50 with a second layer of Eco10.

The fully constructed G-Bots (including the ring magnets) were tested for stress and strain using a CellScale UniVert Universal Testing system (Figure S3). In the compression testing mode, the Ecoflex body was placed on a stage where a block, attached to the force load, would be programmed at a certain speed to move downwards until it had compressed the G-Bot body to 20% of its length. The block/force load’s initial position was 22 mm from the stage. For strain testing, two prongs were used, with one prong fixed to the bottom of the machine and clamped around the bottom of the Ecoflex body. The other prong was clamped on the opposite end of the Ecoflex body and was attached to the force load. The top prong was then brought upwards to stretch the Ecoflex body at 50%, 100%, 150%, and 200% of its original dimensional length with the prong returning to its original position in between each stretch.

Magnetic field and force strength of the magnets were first estimated using COMSOL Multiphysics simulation. The external robot arm magnet was simulated at 5 and 10 mm distance to generate 131 to 260 mT magnetic field strength (corresponding to gradients of 7.3-13.2 T/m). The forces were measured and calculated for all four magnetic rings. These results were then verified with the experimental setup seen in Figure S9, utilizing the robot arm external magnet, the G-Bot in various positions, and an apparatus to measure the magnetic pulling force in the orthogonal direction using a balance (BCE822-1S, Sartorius Lab Instruments GmbH & Co. KG, 37070 Goettingen, Germany). The magnetic force was then calculated for magnetic field strengths of 5 mT to 130 mT (corresponding to gradients of 0.1-7.3 T/m).

#### Orientation control

A seven-degree-of-freedom robot arm (LBR Med 7 R800, Kuka) carrying a permanent magnet was introduced to the completed G-Bot, which was placed and submerged in water. The robot arm’s movement was pathed in various ways to induce various mechanical movements in the G-Bot, as visualized in Figure S6.

The robot arm was placed 15 cm away from the container to create an external magnetic field of approximately 4.1 mT at that distance. The robot arm was then programmed to move from one position to another and back while maintaining this distance. The bot then rotated its magnetic direction. The G-Bot’s movement was observed for these different programmed actions. A GM1-ST DC Gauss-meter (AlphaLab, Inc., Salt Lake City, UT, USA) was used to measure the magnetic field strength at these appropriate distances.

#### Payload delivery and leak tests

The robot arm was placed at a certain distance away from the edge of the container to create a certain external magnetic field strength. A syringe was used to introduce water to the G-Bot inner cavity. After the G-Bot was deemed fully compressed, the volume of water remaining in the G-Bot was measured to determine the volume that was delivered from the original capacity.

Different accordion models fabricated from the different Ecoflex material body types were tested at 173 mT, 110 mT, and 40 mT (measured/verified using Gaussmeter) to examine differences in delivery with different external magnetic field strengths (Figure 3). The magnetic field was introduced until there was full compression of the G-Bot or 30 minutes had passed, after which, the remaining liquid payload in the G-Bot was measured. After, the accordion and concertina models (made of Ecoflex 10-50 bodies) were compared for payload efficiency using the same methodology.

A leak test was done to verify the mechanism of payload release was predominantly compression. 0.75 mL of 0.01 mg/mL methylene blue mixture was loaded into the G-Bot, which was then placed in a 50 mL beaker filled with DI water and agitated on an orbital shaker at 50 rpm (Figure 3E). At 30-minute intervals over a 5-hour period, a 300 μL liquid sample was collected from the beaker and transferred to a NUNC 96-well plate. The resulting absorbance values were observed at a wavelength of 665 nm and compared to a standard curve. The resulting concentrations and payload leakage from the G-Bot were calculated.

#### Operating C-arm for delivery, sampling and locomotion

The G-Bot was examined for visibility under clinical X-ray fluoroscopy using OEC 3D C-arm (GE). Its clinically relevant X-ray attenuation was validated by comparing its relative contrast with three different syringes containing (1) DI water (2) 30wt.% aqueous iohexol solution and (3) 100wt.% aqueous iohexol solution (Figure 4A). A gray-scale analysis was done to compare visibility of the G-Bot to the three samples (Figure 4B).

To demonstrate liquid sampling, the G-Bot was placed into a container of 100wt.% iohexol (Figure 5A). The G-Bot was given a payload of 0.7 mL of deionized water to ensure it was submerged in the contrast environment (Figure 5B). X-ray fluoroscopy was used to visualize the G-Bot before being placed in the liquid environment. The robot arm introduced a magnetic field to ensure full compression of the G-Bot, and then the polarity was shifted to allow for expansion of the G-Bot (Figure 5A). The G-Bot post-sampling was analyzed with and without its capsule under X-ray fluoroscopy.

To investigate viscous sampling, a 250 mL solution of 50% deionized water and 50% iohexol was made to create a 2.3wt% and 4.5wt% gelatin mixture (Figures 5C and 5D). The empty G-Bot was submerged into the environment with the robot arm magnetic field being introduced for full compression of any air bubbles and then expansion. X-ray fluoroscopy was used to visualize the G-Bot before and after the compression/expansion. Any sample remaining in the G-Bot was taken up by a syringe for measurement and then visualized again under X-ray fluoroscopy. This was repeated three times for each of the gelatin solution weight percentages. For the 4.5wt% solution, the DICOM image from the X-ray fluoroscopy was visualized in a 3D Slicer program and then recreated in Fusion360 to analyze the subsequent volume taken up by the G-Bot.

In Figure 6D, 7-inch silicon tubes were arranged to create a phantom colon model. The tube was then submerged in an opaque gelatin solution (6wt.% gelatin). The G-Bot is then introduced into the system containing a liquid payload. An external magnetic field from the robot arm was used to manipulate the G-Bot along the model. X-ray fluoroscopy was used throughout to visualize the position of the G-Bot. Once the bot was positioned at the end of the tube, a larger external magnetic force was introduced for G-Bot compression. X-ray fluoroscopy was used to confirm the payload release and G-Bot compression.

### Quantification and statistical analysis

All quantitative data are represented as mean ± standard deviation (SD). Independently fabricated G-Bots were considered the experimental units, while repeated measurements were treated as technical replicates. The reported sample size (n) reflects the total number of measurements, with the number of independent G-Bot samples specified in the corresponding figure legends. Unless otherwise is noted, measurements were performed using 2-6 independently fabricated G-Bot samples, with multiple technical measurements (10 repetitions per sample) conducted under comparable conditions For comparisons involving multiple groups, statistical significance was assessed using one-way analysis of variance (ANOVA). For pairwise comparisons, a two-tailed Student’s t-test was used. A p value < 0.05 was considered statistically significant. Across the manuscript, ∗∗∗∗ denotes a p value < 0.0001, ∗∗∗ denotes a p value between 0.001-0.001, ∗∗ denotes a p value 0.001-0.01, and ∗ denotes a p value 0.01 to 0.05. Statistical analyses were performed using GraphPad Prism 10.0.0.
