# Supplementary material for: Fluoroscopic control of a magnetorobotic capsule for precision gastrointestinal sampling and delivery
Source: iScience. 2026 Feb 11;29(3):115007. doi: 10.1016/j.isci.2026.115007 (PMC13080475; doi:10.1016/j.isci.2026.115007)
Supplement: Document S1. Figures S1–S9 and Data S1 [file mmc1.pdf]

iScience, Volume *29*

Supplemental information

Fluoroscopic control of a magnetorobotic capsule for precision gastrointestinal sampling and delivery

Sophie Nguyen, Tuan-Anh Le, Melek Naz Guven, Carol Lu, Husnu Halid Alabay, and Hakan Ceylan

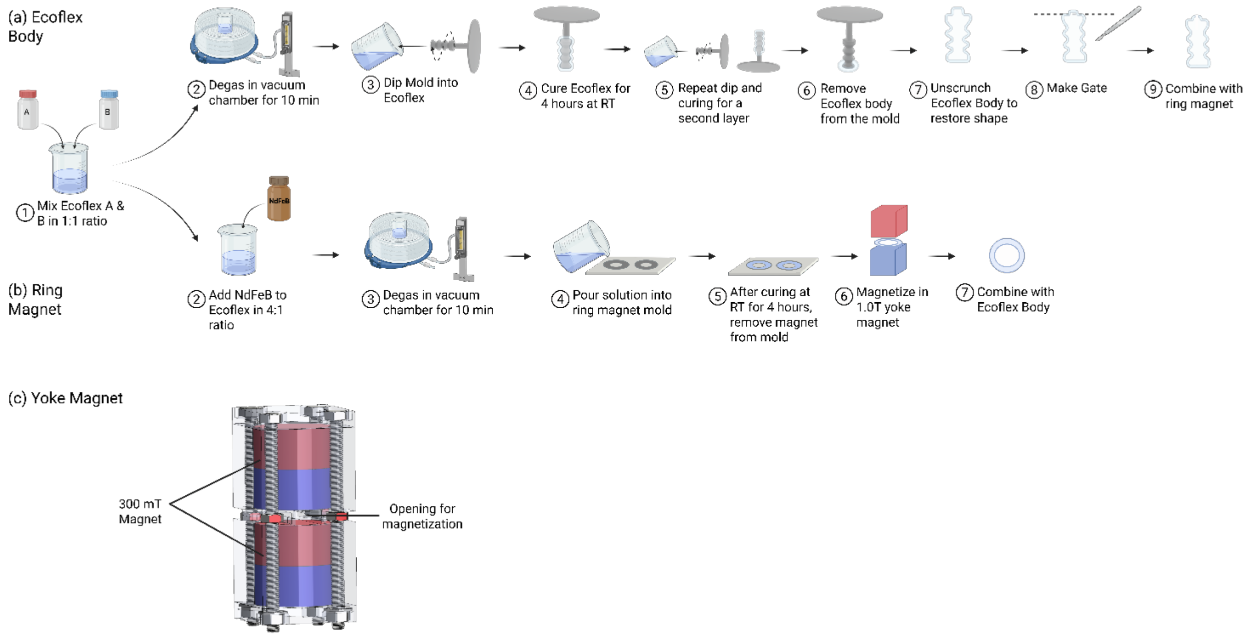

Figure S1: Fabrication protocol for the soft molded parts of G-Bot. (a) Ecoflex body fabrication using Ecoflex silicon polymer materials and a 3D-printed mold. (b) Ring Magnet fabrication that utilizes Ecoflex silicon polymers, NdFeB particles, and a 3D-printed mold. (c) Model of the yoke magnet containing two 300 mT magnets and an entrance to slide soft robotic parts in for magnetization.

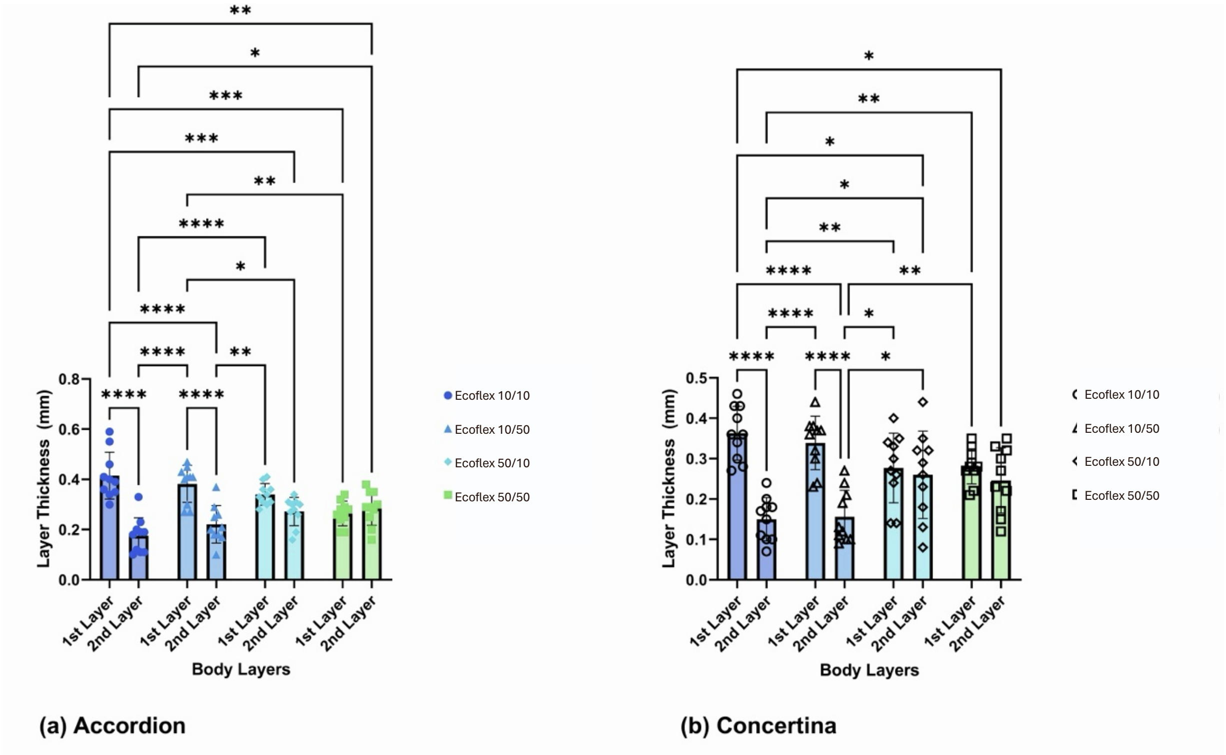

Figure S2: Comparative analysis of the inner capsule compositions. Body layer thickness comparison for different (a) Accordion and (b) Concertina geometries and double-walled Ecoflex body constructs. For both geometries, n = 10. (**** denotes a p value < 0.0001, *** denotes a p value between 0.001-0.001, ** denotes a p value 0.001-0.01, and * denotes a p value 0.01 to 0.05.)

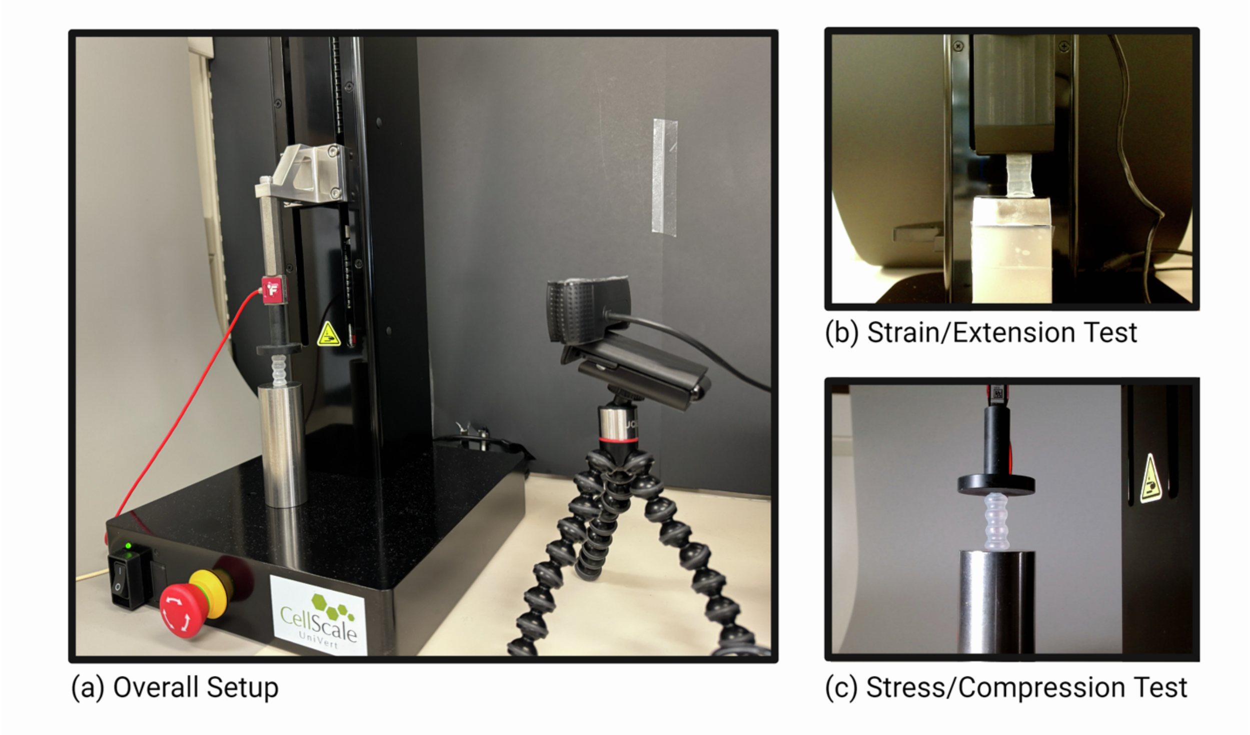

Figure S3: Experimental setup for the compression testing involving the UniVert testing system, a force load, and a camera. Clips with the Ecoflex body fixed on one end were used for the strain testing.

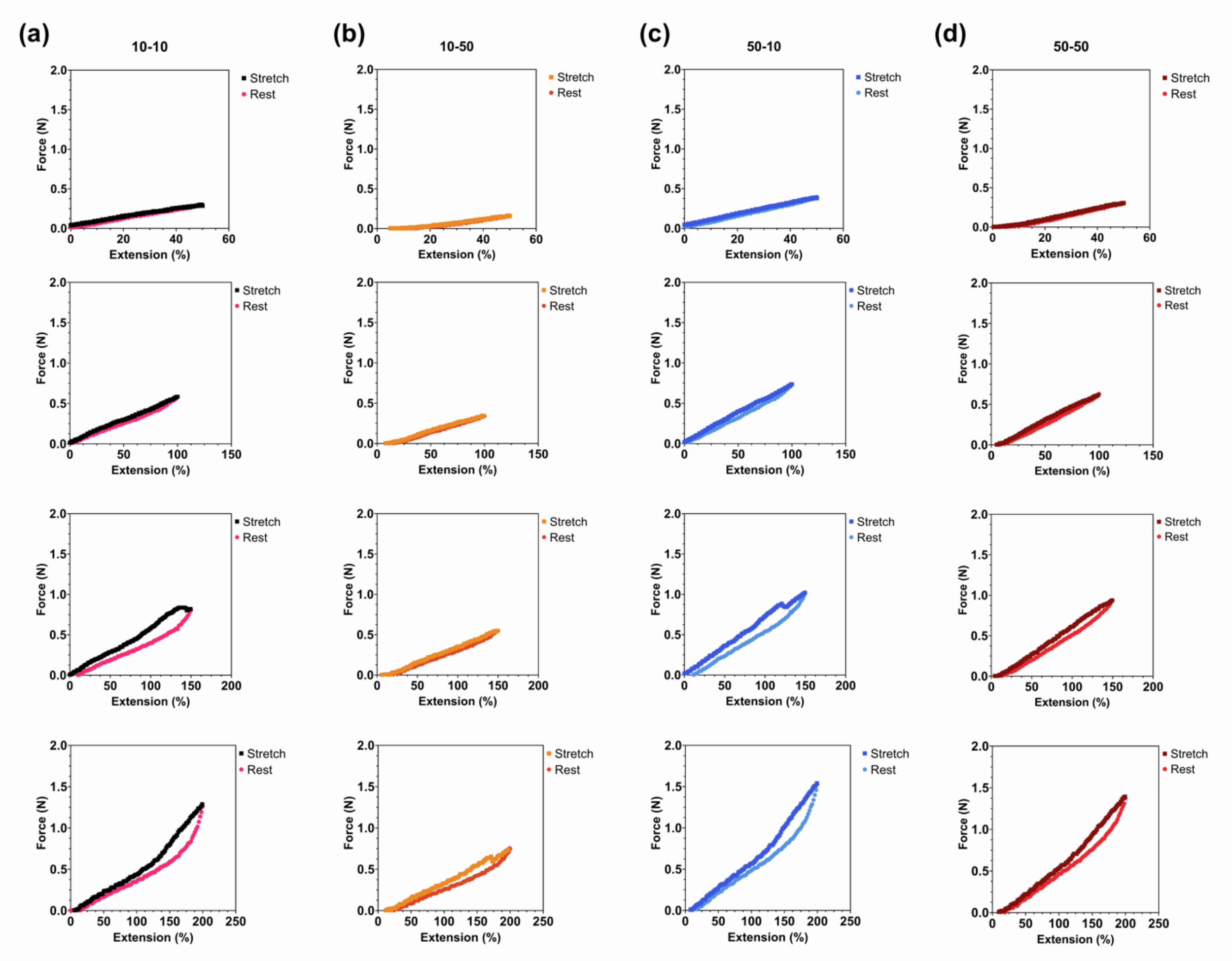

Figure S4: Mechanical response (strain-stress curves under extension) of G-Bot inner capsules with different material compositions. Strain and recovery curves for different Ecoflex body construct types at a 50%, 100%, 150%, and 200% extension of its normal body length (20 mm). Results are shown for (a) Eco10/10, (b) Eco10/50, (c) Eco50/10, and (d) Eco50/50. For all the body constructs, n = 5.

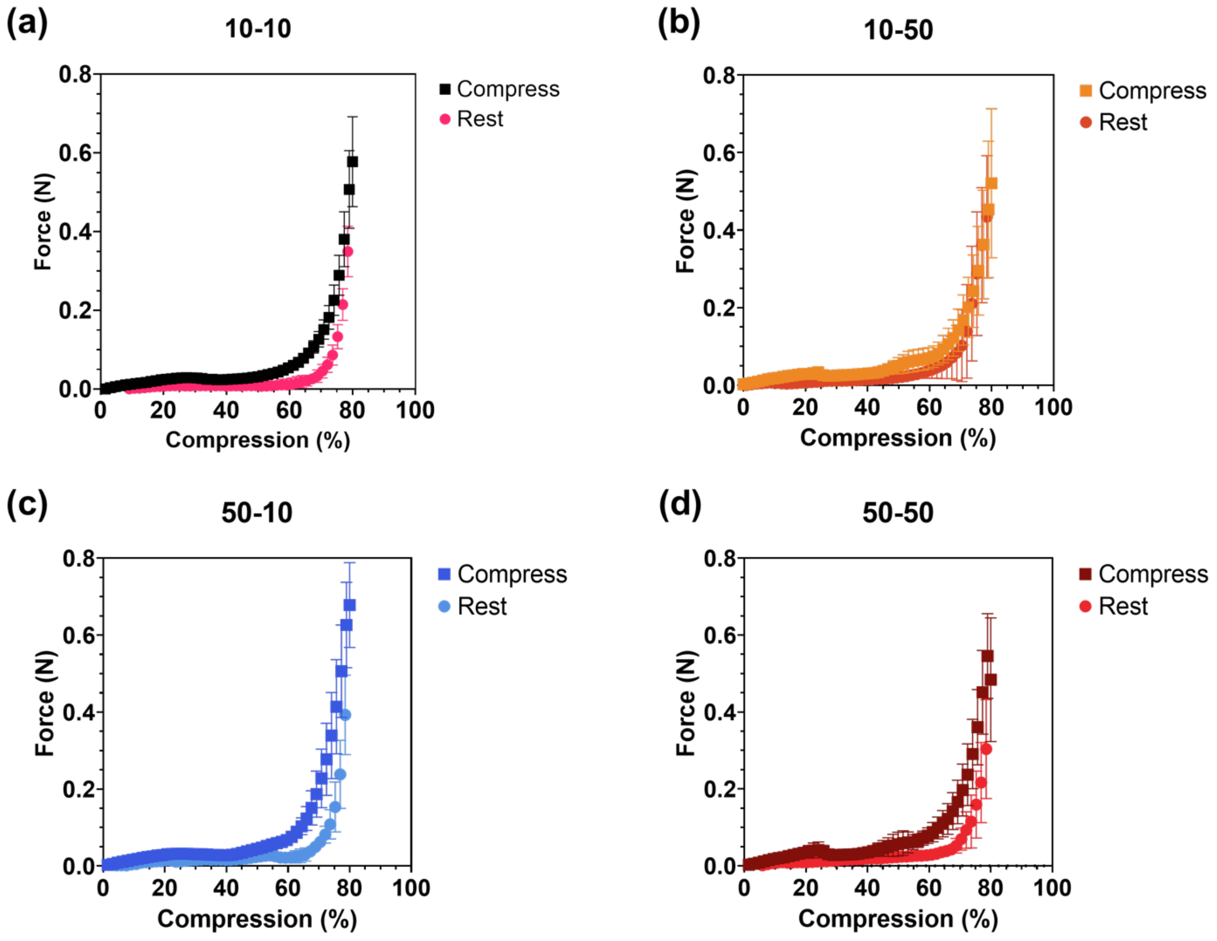

Figure S5: Forces required to stretch or compress G-Bot inner capsule. Compression and recovery curves for different Ecoflex body construct types at 80% compressive displacement. Results are shown for (a) Eco10/10, (b) Eco10/50, (c) Eco50/10, and (d) Eco50/50. For all the body constructs, n = 5 with 5 repeated measurements per sample.

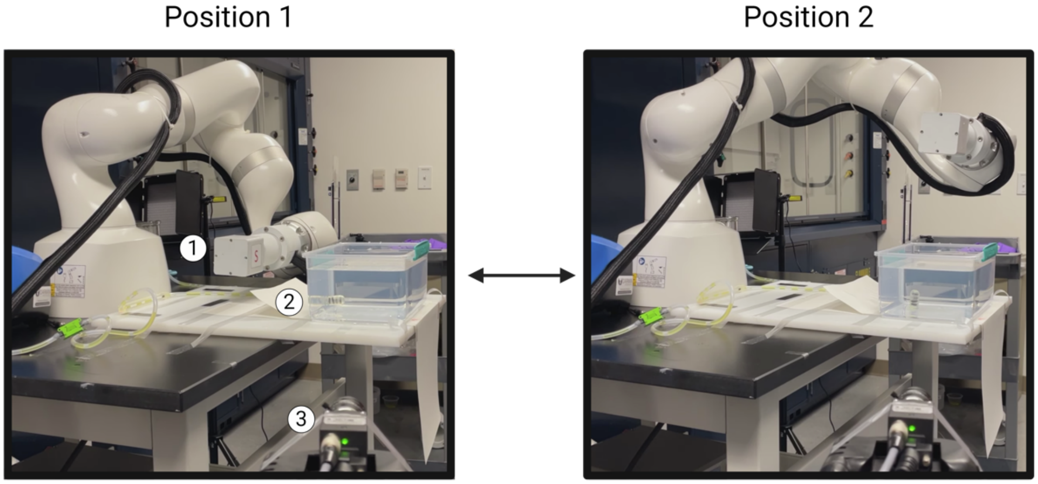

Figure S6: External magnetic field control using the robot arm carrying a permanent magnet at its tip. Experimental Setup for testing the orientation control involving a (1) robot arm to create the external magnetic field, (2) the G-Bot submerged in a container of water, and (3) a camera to capture the movement. The robot arm was placed in two different positions to evaluate orientation control along two axes.

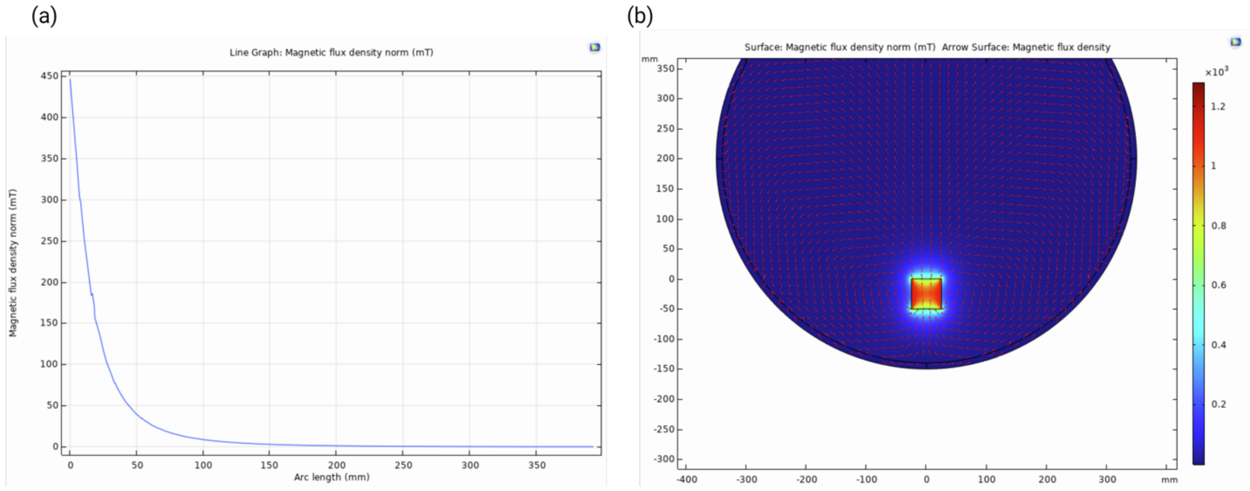

Figure S7: Visualization of the external magnetic field of the robot arm magnet as a relation of distance from the magnet using COMSOL Multiphysics. (a) Magnetic field strength based on a linear distance from the magnet surface. (b) Magnetic strength and direction in relation to the magnet in a 2D plane.

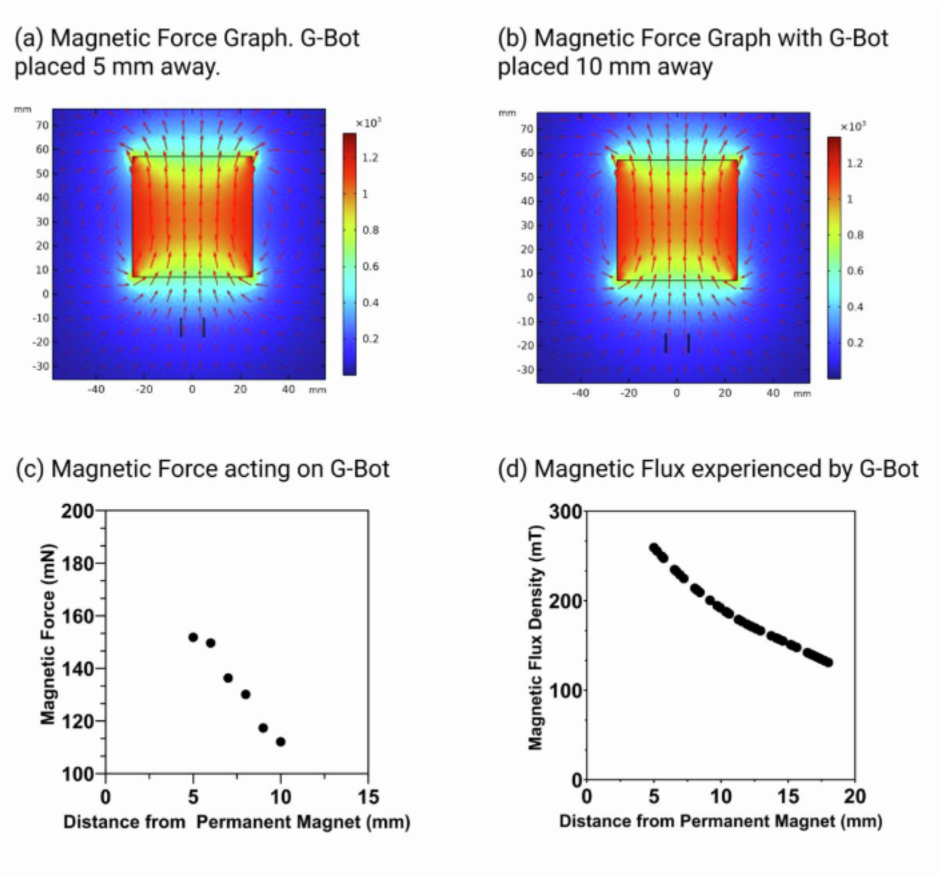

Figure S8: Visualization of the magnetic forces acting on the G-Bot at various distances from the external permanent magnet and various external magnetic field strengths.

Data S1. Calculation of the magnetic moment for the ring magnet geometry.

Volume of one ring:

$$V = \pi \left(\frac{10\ mm}{2}\right)^2 \times 2\ mm - \pi \left(\frac{9\ mm}{2}\right)^2 \times 2\ mm = 29.85\ mm^3 = 2.98 \times 10^{-8}\ m^3$$

Volume of NdFeB in one ring for a ratio of 1:4 Ecoflex:NdFeB

$$V_{\mathrm{NdFeB}} = 0.363 \times 2.98 \times 10^{-8} = 1.08\ \times 10^{-8}\ m^3$$

Magnetization of NdFeB under a ~1 T (~764 kA/m) is approximately 0.47 T (374 kA/m).

Magnetic moment of one ring magnet is:

$$m = \frac{1}{\mu_0} B_r V = \frac{0.47T\ \times 1.08 \times 10^{-8} m^3}{4\pi \times 10^{-7} H/m} = 4.04 \times 10^{-4} A/m^2$$

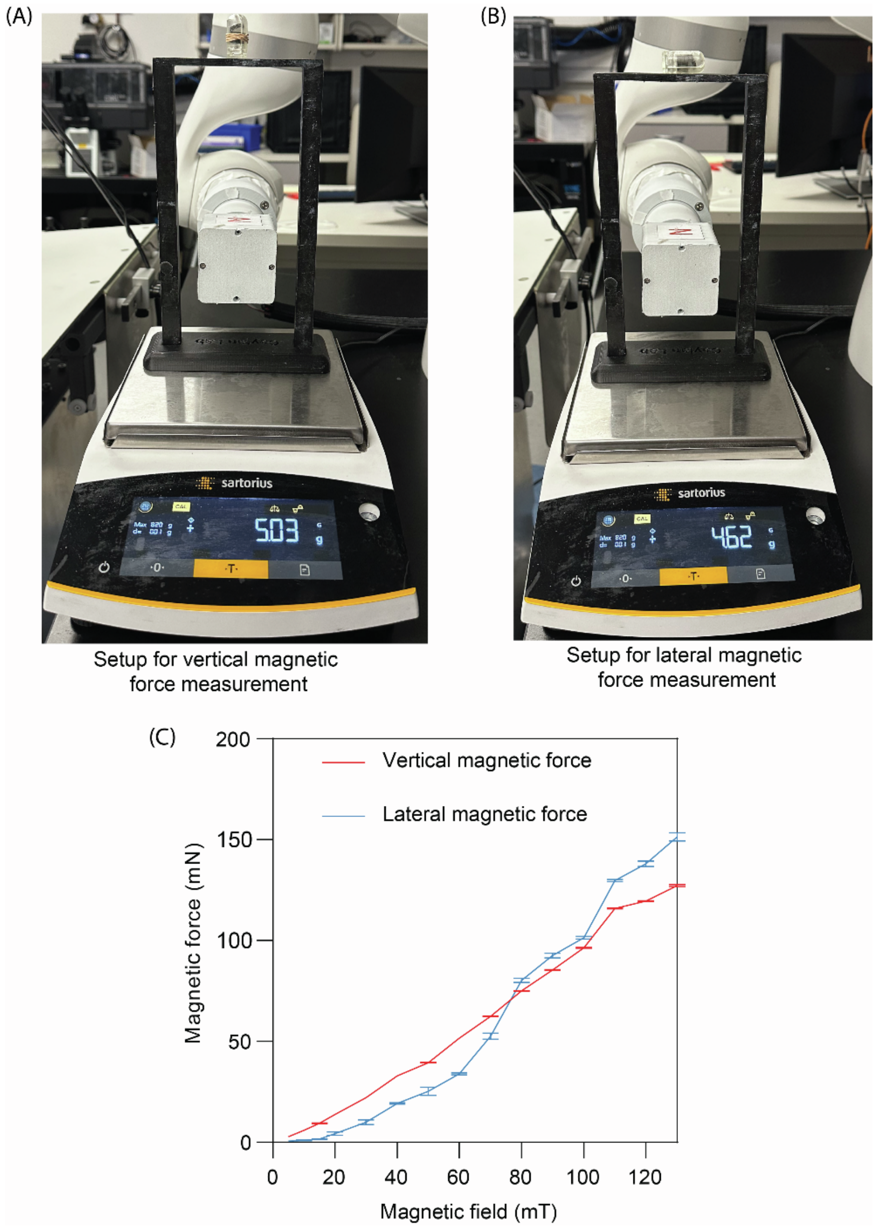

Figure S9: **Measurement of the magnetic force of the G-Bot in both vertical and lateral configurations.** (a) Experimental setup for the vertical magnetic force measurement with the G-Bot in the vertical position, the robot arm external magnet, and the external frame. (b) Experimental setup for the lateral magnetic force measurement of the G-Bot. (c) Graphical illustration of the magnetic force of the G-Bot for both configurations from 5 mT to 130 mT external magnetic field.
